# The efficacy of a low-sodium salt substitute enriched with potassium to improve sodium-to-potassium ratio and reduce blood pressure in adolescents and their families in Soweto, South Africa: study protocol for randomised controlled trial

**DOI:** 10.1186/s13063-025-09184-z

**Published:** 2025-11-04

**Authors:** Simone H. Crouch, Lisa J. Ware, Shane A. Norris, Aletta E. Schutte

**Affiliations:** 1 MRC/Wits Developmental Pathways for Health Research Unit, Department of Paediatrics, Faculty of Health Sciences, University of the Witwatersrand, Johannesburg, South Africa; 2 School of Human Development and Health, University of Southampton, Southampton, UK; 3 The University of New South Wales, The George Institute for Global Health, Sydney, Australia; 4 Hypertension in Africa Research Team (HART), MRC Unit for Hypertension and Cardiovascular Disease, North-West University, Potchefstroom, South Africa

**Keywords:** Salt substitute, Blood pressure, Sodium, Potassium, Clinical trial, Africa

## Abstract

**Introduction:**

The burden of cardiovascular disease in Sub-Saharan Africa has increased in recent years, and high blood pressure is the leading cause. One established risk factor for hypertension and cardiovascular disease is dietary salt intake. The World Health Organisation has highlighted low-sodium salt substitutes (LSSS) as a potential method to lower sodium intake. LSSS enriched with potassium may additionally support improving sodium-potassium balance. Studies in India and China have investigated the impact of LSSS on reducing sodium intake and the risk of stroke and hypertension in adults. However, evidence in African populations, and in particular youth, is lacking. As such, this protocol describes a phase 1 double-blinded randomised controlled trial to assess the efficacy of a potassium-enriched LSSS compared to traditional salt to improve urinary sodium-to-potassium ratio and blood pressure in African adolescents and their families.

**Methods:**

We will enrol 600 adolescents (13–19 years old) and their primary caregivers living in Soweto, South Africa. Adolescents and their households will be randomised to receive a LSSS or traditional table salt (NaCl) for a 16-week period. All other household salt products will be removed. Anthropometrics and questionnaire data will be collected at 0 and 16 weeks. Spot urine samples and blood pressure will be collected at 0, 4, 12 and 16 weeks. Safety screening for kidney function will be conducted on household members at baseline. The trial protocol received ethics approval from the University of Witwatersrand Medical Human Research Ethics Committee (M221056).

**Discussion:**

The obtained results will, to the best of our knowledge, be the first in an African population to provide insights into the efficacy of a potassium-enriched LSSS in improving urinary sodium-to-potassium ratio and blood pressure.

**Trial registration:**

This trial is registered with the Pan African Clinical Trials Registry (https://pactr.samrc.ac.za); identifier: PACTR202306727520808 (09 June 2023).

**Supplementary Information:**

The online version contains supplementary material available at 10.1186/s13063-025-09184-z.

## Introduction

Bloodpressure (BP) levels in Africa have risen steadily and are now among the highest in the world [1]. As such, high BP is an increasingly important cause of death and disability in Africa. Dietary sodium intake is an established risk factor for hypertension and cardiovascular disease [2–4]. Despite South Africa being one of the few World Health Organisation (WHO) member states with mandatory sodium reduction policies in place, a 2023 WHO report found South Africa and the African region off track to meet the 2025 or 2030 sodium reduction targets [5]. In this report, the WHO recommended research and innovation specifically to investigate low-sodium salt substitutes (LSSS) as a method to lower sodium intake in populations where intake is high from discretionary use of salt added in cooking and prior to eating. In the Salt Substitute and Stroke Study (SSaSS) in China, salt substitutes reduced the risk of major adverse cardiovascular events (rate ratio, 0.87; 95% CI 0.80 to 0.94; *P* < 0.001), stroke (rate ratio, 0.86; 95% CI 0.77 to 0.96; *P*= 0.006), and led to a reduction in BP (mean difference in systolic BP; − 3.34 mm Hg (95% CI − 4.51 to − 2.18) over 5 years [6]. Similar BP reductions were shown in a salt substitute trial in India where SBP (mean difference, −4.58 mmHg, 95% CI: −6.20, −2.97 mmHg, *P* < 0.001) and DBP (−1.14 mmHg, 95% CI: −2.13, −0.15, *P*= 0.02) were lower in the intervention group [7]. Recognising the growing body of evidence for LSSS efficacy to lower BP in adults from multiple populations, including a Cochrane Systematic Review [8], the WHO launched an online public consultation on the draft guideline on the use of LSSS. Key gaps identified through this consultation process included (i) lack of data from Africa and (ii) the lack of impact data of LSSS at the household level, including for children. In addition to the effect LSSS may have in reducing sodium, mounting evidence supports the importance of a sodium–potassium balance in reducing BP [9, 10]. Evidence has shown that the sodium-to-potassium ratio is a better indicator of BP outcome than either sodium or potassium alone, and this was seen with both 24 h [11, 12] and spot urine collection [13–16]. Spot urinary sodium-to-potassium ratio has also been found to associate with the risk of stroke [17]. With the high participant burden of 24-h urine collections (often suffering from incomplete collections [15]), and the strong evidence of an association between spot urinary sodium-to-potassium ratio and BP [13–16], spot urine sodium-to-potassium ratio serves as an effective and convenient indicator of BP outcome. Sodium-to-potassium ratio has been found to show diurnal variations [16], furthermore, urinary sodium and potassium levels in second-morning urine more closely correlate to 24-h urine than that in first-morning urine [18]. As such controlling the time of sample collection to the second urine sample in the morning will result in a reduction in variability and more closely correlate to 24-h urine.

To investigate perceptions towards LSSS enriched with potassium in a South African context, we engaged in community discussions followed by taste and visual tests comparing various commercially available LSSS products enriched with potassium with usual salt products (100% sodium chloride—NaCl) in Soweto, South Africa. From the various LSSS products tested (35%, 50%, 66% potassium chloride—KCl blends and 100% KCl), this study found that almost half of the participants ranked the taste of an LSSS enriched with potassium (50% KCl, 50% NaCl) as “fantastic or really good” and most “liked and would be happy to use it” or felt it “tasted like common salt”—suggesting a 50% KCl LSSS will be well tolerated in a South African population [19]. Accurate visual identification of various LSSS products compared to 100% NaCl was found to be generally low [19]. Given the limited information available on LSSS in African and child populations, data are urgently needed to examine the impact of LSSS on sodium reduction and BP across multiple age groups of South Africans. As such, we hypothesised that replacing household table salt with a 50/50 KCl/NaCl LSSS will improve the spot urinary sodium-to-potassium ratio of both adolescents and adults and will reduce systolic BP in adults.

### Aim

The primary aim of the trial is to assess in African families the efficacy of a LSSS enriched with potassium compared to usual salt in improving the spot urinary sodium-to-potassium ratio and lowering BP over four months.

## Methods

### Public engagement in trial development

Formative research conducted in Soweto communities influenced two elements of the SALT Trial protocol. Target population: Focus group discussions were conducted with young adults in Soweto on the perceptions of and potential barriers to the use of LSSS. The results highlighted the need for the LSSS to be well explained at a household level to avoid individual household member fears or misunderstandings dissuading use by others. As such, the trial focuses on both adolescents and primary caregivers, and where the primary caregiver is under 40 years of age, an additional older household member is included to ensure wider household inclusion, increasing potential acceptability. Furthermore, the involvement of the whole household in kidney screening ensures LSSS are fully explained to all household members irrespective of enrolment in the trial. Intervention product: Focus group discussions also highlighted taste as a significant barrier to LSSS acceptability. As such, we conducted taste and visual tests comparing various LSSS products enriched with potassium with usual salt products in the target community. This study found 50/50 KCl/NaCl LSSS ranked highest among participants [19]. As such, the 50/50 KCl/NaCl blend was selected. The low accurate visual identification of various LSSS products indicated a low risk of participant visual differentiation of products, and as such, unmarked salt flask packaging was sufficient to ensure product concealment.

### Trial design and procedures

**Fig. 1:**
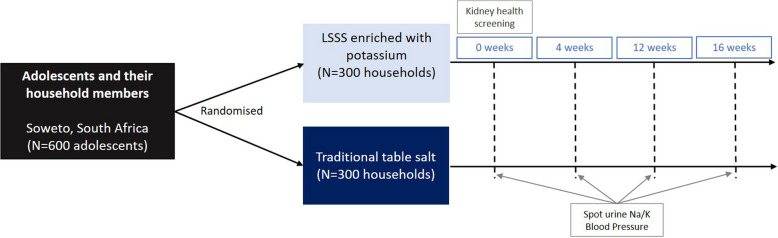
Trial design

The SALT study is a two-arm double-blind randomised controlled superiority trial to be conducted in Soweto, an area of historical disadvantage in South Africa (Fig. 1).

### Phase 1 trial design

This trial utilises a 50% LSSS, which was determined to be acceptable from a visual and taste perspective by a panel of adults living in Soweto [19]. However, previous trials have typically tested lower doses of potassium; for example, the SSaSS trial in China used a 25% potassium LSSS [6]. As such, regular safety monitoring with adverse event tracking will be employed. Furthermore, the trial recruits households from the general population (i.e. not a patient population). This includes adolescents and their caregivers, providing novel information on the use of LSSS in a paediatric population.

This aligns with our adaptive Phase 1 design and objectives to investigate safety before progressing to later efficacy-focused phases. The inclusion of preliminary efficacy outcomes is used to indicate the feasibility of household salt supplementation within an African urban setting. This adds further value to this early Phase 1 investigation to understand participant behaviours, including salt use and food consumption outside of the household that may influence outcomes. The results of this Phase 1 trial will inform the design of a subsequent Phase 2 efficacy trial, including refinement of sample size calculations and outcome selection.

Furthermore, incorporating acceptability, feasibility, safety, and exploratory efficacy in one trial design holds the potential to accelerate the translation of the results, of critical importance in the South African setting where hypertension is prevalent and control remains a challenge [20]. Lastly, within this resource-constrained setting, this adaptive Phase 1 trial design maximises efficiency and increases the ethical value of the data generated through participants’ contribution to better understanding the potential health benefits within this context.

### Participants

#### Inclusion criteria

The study will include adolescents, aged 13–19 years, male or female of African descent living in Soweto. Soweto is located in the South West of the City of Johannesburg, covering 200 km^2^ with over 1.8 million people (approximately 9000/km^2^). The study will also include the adolescent’s primary caregiver (parent/guardian) aged ≥ 40 years, and where the primary caregiver is < 40 years of age, an additional member of the household (aged 40–70 years) will be included. All included household members should eat and stay in the home at least five days a week. Individuals presenting with new clinical hypertension will be included but referred. Those using BP-lowering medications, specifically angiotensin-converting enzyme inhibitors or angiotensin receptor blockers, will be included.

#### Exclusion criteria

Households where any household member is unable to read or understand English, self-reports any previous diagnosis of or test results showing kidney disease/diminished kidney function, use of potassium-sparing diuretics or potential contraindication to the LSSS, will be excluded. Households with individuals considered to eat most meals outside of the home, with a previous diagnosis or currently self-reporting any eating disorder, participating in any other trial, high-level athletes, self-reported pregnancy at the time of screening, or households without cooking facilities, will be ineligible.

### Supply of salt products

At the start of the trial, all salt in the household will be removed and weighed by the study team; the equivalent weight in salt will be returned to the household at the end of the trial. The trial will have a 16-week duration. Both participants and investigators will be blinded to the intervention. The LSSS and normal table salt (control) will both be supplied in identical unmarked salt flasks to that used commercially by Cerebos (the largest salt supplier in South Africa). Households will be contacted regularly to ensure a sufficient supply of salt products and encourage compliance. Participants will be given a number to contact should they be running low on the provided product. No provisions will be made for continued access to either trial product following completion of the trial.

### Intervention

Adolescents and their households will be provided with a LSSS. The LSSS will be a blend of 50/50 KCl/NaCl manufactured by Cerebos (Cerebos Ltd, South Africa).

### Control

Adolescents and their households will be provided with normal table salt. The table salt will be 100% NaCl, manufactured by Cerebos.

### Outcomes

The primary outcome will be the change in spot urinary sodium-to-potassium molar ratio from baseline to 16 weeks in adolescents. Secondary outcomes include the following: (i) change in spot urinary sodium-to-potassium molar ratio in adults (caregivers and additional household members); (ii) change in systolic and diastolic BP in adults; (iii) change in systolic and diastolic BP in adolescents.

### Sample size

A sample size of 470 will be sufficient to detect as statistically significant at the 5% level a 0.6 change in sodium-to-potassium ratio at > 90% power [21, 22]. A sample size of 488 will be sufficient to detect as statistically significant at the 5% level a 5 mmHg difference in the mean systolic BP of the groups at a power of > 90% assuming a common within-group standard deviation in systolic BP of 17 mmHg [23]. Accounting for a 20% dropout, we will recruit a total of 600 adolescents and their primary caregiver. Where the primary caregiver is younger than 40 years of age, an additional household member aged 40–70 years will be recruited, resulting in a total study population of 1200–1800 (600 adolescents and 600–1200 adults).

### Risks to the safety of participants

The study protocol received ethics approval from the University of Witwatersrand Medical Human Research Ethics Committee (HREC) (M221056). Written informed consent will be obtained from all study participants before any data collection or other study procedures commence by a research assistant fluent in the participant’s home language. Following engagement with HREC, various clinicians (nephrologists and cardiologists) and a trial-experienced biostatistician, a consensus process was used to develop safety processes. *Screening for safety:* A household composition questionnaire will be administered to the head of the household to determine which individuals compose the household, regardless of familial relationship. All household members will have a screening questionnaire administered to determine self-reported hypertension, self-reported chronic kidney disease and medication use. Participants under the age of 18 years will be asked to collect a mid-stream urine sample into the sterile container provided. Roche Combur test strips will be used to assess glucose, protein, erythrocytes, and leukocytes. Households with a participant showing signs of proteinuria will be excluded, and the participant referred. In participants 18 years and older, a finger prick capillary blood sample will be analysed for creatinine and estimated glomerular filtration rate (eGFR) with a point-of-care device (StatSensor®, Nova Biomedical Corporation, Massachusetts). Where point-of-care eGFR is between 48 and 72 mL/min, venous blood samples will be collected, and serum creatinine will be analysed using a Randox Plus clinical chemistry analyser (UK). Households with a participant showing signs of diminished kidney function (eGFR < 60 mL/min) will be excluded and the participant referred. *Monitoring:* Safety outcomes will be monitored by an independent data safety and monitoring board (DSMB) comprised of a cardiologist, nephrologist and biostatistician. The trial steering committee (TSC) and DSMB will meet throughout the year to review progress, implementation of the protocol, participant safety, any loss to follow-up, guide troubleshooting and review reported adverse events. *Reporting:* Any serious unexpected adverse event (SAE) will be reported to the ethics committee and DSMB within 7 to 15 days. Where indicated, participants will be directly linked to care by the trial manager, and not simply issued a referral letter. Whether or not the adverse event is considered to be related to the trial will be indicated on a form submitted to the HREC. Should an adverse event require a participant to be withdrawn from the trial, the full household will be withdrawn from the trial. Should a participant suffer harm due to their participation in the trial, they will receive the appropriate care and services required according to their state of health. All adverse events, irrespective of type, will be presented descriptively (AE term, onset time, allocation group, severity, AE outcome, relationship to the study). *Participants* < *18 years:* Should any participant below 18 years of age report abuse, or a research team member suspect abuse of a minor, whether physical or sexual, this will be reported to an appropriate child protection agency or Department of Social Development as legally required. *Confidentiality:* Participants will be assigned a unique study number for all study information and labelling of results. No personal identification will be displayed in any reports or communication without the specific consent of the participant.

### Protecting against sources of bias

At randomisation, concealment allocation will be strictly observed. All trial staff and researchers will be blinded to intervention or control group allocation (double-blind trial design). Trial product is stored in identical unmarked packaging labelled A or B on the bottom of the flask. Unblinded allocation of intervention and control products is known only to an independent team not involved in the trial.

### Study procedures

*Recruitment into the trial:* Adolescents screened for previous clinical trial, but not qualifying, who indicated willingness to be contacted about future research were approached for the SALT trial. Research staff will visit households and invite adolescents and their primary caregiver to participate in the trial. Verbal and written information will be provided in their preferred language, and they will have the opportunity to ask questions. Consent will be obtained from all participants over 18 years. For participants under 18 years of age, informed assent will be obtained from the participant following informed consent from a legal guardian. Consent and assent will also be obtained from all household members for safety screening. *Screening for risk:* All individuals residing within the household of eligible participants will be screened for safety. *Baseline assessment and randomisation:* Eligible consenting adolescents and their primary caregivers will then enter the trial and undergo baseline assessments in their homes. Thereafter, they will be randomised to receive either the intervention or control product. Participants will be visited again at weeks 4 and 12 to ensure sufficient supply of trial product and conduct monitoring assessments. *Exit assessment:* Adolescents and their primary caregivers will undergo exit assessments in their homes at week 16. *Process evaluation:* Will be conducted with participants throughout the trial. *Retention:* To facilitate participant retention, several strategies will be implemented. Regular contact will be maintained through telephone calls, SMS, and WhatsApp messaging. At each home visit, participants will be asked to verify and update their contact information and, where applicable, to provide additional contact numbers. Study staff will also provide participants with contact details for the research team, enabling them to communicate changes in their information between scheduled visits. To acknowledge participants’ time and contribution, financial reimbursement will be provided at both the initiation and conclusion of the trial.

### Randomisation

Baseline data collection will be completed on all eligible participants meeting the inclusion criteria. Adolescents (and their households) will be randomized in a 1:1 allocation ratio to group A or B using block randomisation. Randomisation is supported by a team independent of the trial and carried out using boxes. Product A and B salt flasks will be batched into sets of 40 with an equal distribution of product A and B (20 flasks of each product), packed in random order into boxes and sealed in a 1:1 ratio. As 600 households will be randomised into the trial, 16 boxes of 40 will be prepared to preserve randomisation. The team conducting box randomisation will be blinded. Based on work from previous trials conducted within the target community, the need for a feeling of choice by participants in the randomisation process is paramount. As such, to ensure this need is met while preserving randomisation, the participant will select a salt flask from the prepacked boxes labelled as either A or B on the bottom of the bottle (not visible during selection). When only one flask remains in the box, these flasks will be placed randomly into a box until full and then sealed and used for the next round of participant randomisation. All members of the randomisation, data collection, analysis and research teams are blinded to the product allocated for groups A and B.

### Study measures

Incoming questionnaires will be administered to obtain demographic and health information including age, sex, ethnicity, hypertension status and related medication use, social vulnerability index [24] (adapted from the Centre for Disease Control social vulnerability index [25]), sleep [26], physical activity [27, 28], food intake [29], and document where salt is stored in the household and in what form of packaging at 0 weeks. At 16 weeks, a questionnaire will be administered to assess perceived salt use [30]. Trained researchers will measure height and weight in triplicate to the nearest 0.1 cm and 0.1 kg using a portable stadiometer (Charder HM200P Portstad; Charder Electronic Co. Ltd, Taichung City, Taiwan) and electronic scale (Omron Healthcare, Kyoto, Japan) in all participants on the same day as the first and last sample collection [31]. Waist circumference will be measured in triplicate to the nearest 0.1 cm following standard measurement protocols [32]. Participants will be asked to collect four mid-stream urine samples into the sterile containers provided (0, 4, 12 and 16 weeks). As the sodium-to-potassium ratio has been found to show diurnal variations [16], and the urinary sodium and potassium levels in second-morning urine more closely correlate to 24-h urine than those in first-morning urine, participants will be asked to collect the second urine sample in the morning [18]. Written and verbal instructions on how to collect a mid-stream urine sample into the sterile container will be provided. Participants will be given a standard plastic specimen container, and collected samples will be kept on ice. Samples will be aliquoted and stored at − 80 °C until the time of analysis. Samples will be analysed for sodium and potassium using the Randox ISE equipment (Randox Laboratories Ltd, Crumlin, UK). Urinary molar sodium to potassium ratio will be calculated for each participant as sodium (mmol/L)/potassium (mmol/L). Three BP and heart rate measurements will be collected from the seated and rested participants on the left arm with a 30-s interval in-between following the International Society of Hypertension measurement guidelines [33] at 0, 4, 12 and 16 weeks using an Omron HBP-1300 BP device (Omron Healthcare, Kyoto, Japan) validated for use in adults and in children [34]. The first measure will be discarded, and the second and third averaged for analysis. Should the second and third measures differ by more than 5 mmHg, a fourth measurement will be taken (Supplementary Material 1).

### Process evaluation

A questionnaire assessing self-reported compliance will be conducted at 4, 12, and 16 weeks. Additionally, at week 16, a questionnaire on perceptions of which group each participant was in will be administered. Interviews will be conducted to ascertain the opinions of participants in the trial and determine perceptions relating to the salt substitute and the feasibility of its use long term in Sowetan households. All interviews will be audio-recorded. Interviews will be conducted in person with 30 adolescents, 20 from the intervention group and 10 from the control group, to determine perceptions of the LSSS as well as willingness and barriers to adopting the LSSS on a permanent basis. Interviews will be conducted within six weeks of the trial ending. Following the first half of the interview, the primary caregiver will be invited to join, and a duo interview will be conducted. The compliance and perception questionnaires as well as the interview guide were developed based on learnings from community feedback during focus group discussions conducted as part of formative work, as well as the learnings of community healthcare workers experienced in research within Soweto. Implementation fidelity, the extent to which the trial is delivered as planned and the quality of the various components, will be assessed via monitoring data informing dose delivered.

### Qualitative data analysis

Audio-recordings from interviews will be transcribed verbatim, and the transcripts compared with the respective audio-recording to ensure accuracy. Transcripts and field notes will be imported into qualitative data analysis software. Data will be organised into themes, using primarily inductive coding. Using a constant comparative approach, these themes and their sub-themes will be used to produce a coding schema. The relationship between themes and sub-themes will be illustrated in a thematic map. Stages in the analysis will include the following: (a) review of the transcripts; (b) development of a coding scheme to represent emergent themes, supported with verbatim quotations; (c) thematic coding of the transcripts using the coding scheme; (d) development of a thematic map to show a way of viewing the data; and (e) repeating this process (stages a–d) until the thematic map reaches saturation. In order to enhance trustworthiness, ten percent of the transcripts will be double-coded to support the development of the coding scheme [35].

### Data management

Data will be collected electronically and uploaded directly onto the Redcap secure server [36]. All information and files will be kept in locked premises at the SAMRC/Wits-Developmental Pathways for Health Research Unit. All data will be available within 6 months from the end of the trial, and metadata will be made available open access through request to any of the investigators. Samples will be kept for a period of 5 years or as long as the Ethics Committee can agree that the specimens can be maintained securely and continue to have research value. Samples will be destroyed when storage ends; this will be after 5 years unless an ethics committee approves longer storage or asks us to end storage earlier. *Dissemination:* The investigator team will publish the results of this trial in academic journals. All data will be presented as group data rather than individual data. Group results may be disseminated back to participants in the form of a results flyer or community engagement event. In the preparation of publications, we will follow the criteria for authorship recommended by the International Committee of Medical Journal Editors (ICMJE); we do not intend to employ professional writers.

### Statistical analysis

The Statistical Analysis Plan (SAP) is included as an Additional File. In essence, data will be cleaned using standardised protocols, and data analysis will be conducted using appropriate statistical software. Participant characteristics will be described using the relevant distribution statistics. Analyses of the primary outcome will be run in both the intention-to-treat (ITT) and per protocol (PP) populations. ITT analysis set: all randomised participants according to their randomised group, regardless of adherence to the protocol, and excluding those who withdrew their consent. This will be the primary analysis set to assess both preliminary effectiveness and safety. The flow of patients through the study will be displayed in a CONSORT diagram. PP analysis set: all participants in the ITT dataset without major protocol deviation. The major protocol deviations are as follows: households that used study salt for less than 20% of the meals cooked at home; participants that have eaten out more than half of the meals. The PP set will be used to rerun analyses of the primary outcome, including sensitivity analyses. Subgroup analysis will not be performed in the PP set. The between-group comparisons for the primary and secondary outcomes of urinary sodium-to-potassium ratios and systolic BP at the end of the trial will be assessed by linear regression adjusted for baseline BP while allowing for repeated measurements of outcomes. Levels of missing data will be reported. All *p*values will be 2-tailed (alpha = 0.05). Interim analyses will not be conducted as urine samples will be analysed in large batches. Missing data will be examined for the analysis of the primary outcome. We will report the percentage of adolescent participants missing Na/K ratio at baseline and each follow-up visit. We will also report the number of participants included in the complete case analysis (CCA) in the primary analysis, i.e. having all the covariates (e.g. age, sex), at least one of the Na/K ratios at baseline and follow-up visits. If the participants excluded from the CCA are ≤ 5% of all the randomised patients, no multiple imputation (MI) will be undertaken. Otherwise, we will perform MI assuming missing at random (MAR). The imputation model will include the treatment arm, all baseline variables (listed in Supplementary material 1), and available Na/K ratio at baseline and follow-up visits. One hundred sets will be imputed using fully conditional specification with discrete variables imputed using a discriminant function [37]. The imputed data will be analysed using the same model as the one used for the main analysis.

### Trial status

A pilot study was conducted between March 2024 and April 2024. From this study, we found both the process and procedures feasible and acceptable, although we did make one adjustment. The range at which venous blood samples will be collected for lab analysis following point of care eGFR was adjusted from 60–90 mL/min to 48–72 mL/min to better conform with the coefficients of variance of the point of care device in use and alleviate any unnecessary venous blood draws. The NovaMax Pro has a sensitivity of 98.9% and a specificity of 85%. Allowing for an additional 5% variance from the reported specificity (lower of the two) to account for potential population differences, this updated range allows for 20% variance in either direction of the cut-point of eGFR < 60 mL/min. Given these minor adjustments, all enrolled pilot participants were transferred to the main trial, which commenced in May 2024. Completion of trial enrolment is expected by September 2024. Following the pilot of the trial and final edits, the protocol manuscript was drafted and submitted for review. Unfortunately, the review process spanned a 6-month time period. Due to the short nature of the trial, this resulted in the completion of recruitment prior to feedback. Data collection has not yet been concluded, and final trial follow-up data collection is expected by February 2025. *Protocol version* April 2024 (version #2). Any protocol amendments will be communicated to investigators, Human Ethics Research Committees, Trial Steering Committee, Data Safety and Monitoring Board, trial participants, and trial registries.

## Supplementary Information

Supplementary Material 1.

Supplementary Material 2.

## Data Availability

All data will be available within 6 months from the end of the trial, and metadata will be open access through request to any of the investigators.

## Abbreviations

BP: Blood pressure
DBP: Diastolic blood pressure
DSMB: Data Safety and Monitoring Board
eGFR: Estimated glomerular filtration rate
HREC: Human Research Ethics Committee
KCl: Potassium
LSSS: Low-sodium salt substitutes
NaCl: Salt
SAE: Severe adverse event
SAP: Statistical Analysis Plan
SBP: Systolic blood pressure
SSaSS: Salt Substitute and Stroke Study
TSC: Trial Steering Committee
WHO: World Health Organisation
