# Supplementary material for: The efficacy of a low-sodium salt substitute enriched with potassium to improve sodium-to-potassium ratio and reduce blood pressure in adolescents and their families in Soweto, South Africa: study protocol for randomised controlled trial
Source: Trials. 2025 Nov 4;26:468. doi: 10.1186/s13063-025-09184-z (PMC12584445; doi:10.1186/s13063-025-09184-z)
Supplement: Supplementary file 1 — Supplementary Material 1. [file 13063_2025_9184_MOESM1_ESM.docx]

**Statistical Analysis Plan (SAP)**

Sodium in Adolescents and adults Lowering Trial: SALT

**Authors:**

Xiaoqiu Liu^1^, Simone H Crouch^2^, Lisa J Ware^2^, Shane A Norris^2,3^, Laurent Billot^1^, Aletta E Schutte^1,2,4^

**Affiliations:**

^1^ The George Institute for Global Health, Faculty of Medicine, UNSW Sydney, Australia

^2^ MRC/Wits Developmental Pathways for Health Research Unit, Department of Paediatrics, Faculty of Health Sciences, University of the Witwatersrand, South Africa

^3^ School of Human Development and Health, Faculty of Medicine, University of Southampton, UK

^4^ Hypertension in Africa Research Team (HART), MRC Unit for Hypertension and Cardiovascular Disease, North-West University, Potchefstroom, South Africa

Corresponding author: Aletta E Schutte

# List of abbreviations

AE Adverse Event

BP Blood Pressure

CCA Complete Case Analysis

CI Confidence interval

DBP Diastolic Blood Pressure

DiD Difference in Differences

IQR Interquartile Range

ITT Intention-to-treat

LSSS Low Sodium Salt Substitute

Na Sodium

Na/K ratio sodium-to-potassium molar ratio

PP Per Protocol

K Potassium

MAR Missing at Random

MI Multiple Imputation

RCT Randomised Controlled Trial

SAP Statistical Analysis Plan

SD Standard Deviation

SBP Systolic Blood Pressure

# Abstract

The SALT trial is a phase I randomised controlled trial (RCT) assessing the efficacy of a low sodium salt substitute enriched with potassium to improve sodium-to-potassium ratio and reduce blood pressure in adolescents and their families in Soweto, South Africa.

The primary outcome is the change of sodium-to-potassium ratio at week 16 from baseline in adolescents. It will be analysed using a linear mixed model. This statistical analysis plan pre-specifies the methods of analysis for every outcome and key variables collected in the trial. It also includes planned sensitivity analyses including covariate adjustments and subgroup analyses.

**Keywords**: salt substitute, blood pressure, sodium, potassium, clinical trial, Africa, Statistical Analysis Plan

**Contents**

[1 List of abbreviations 2](#_Toc209093064)

[2 Abstract 2](#_Toc209093065)

[3 Administrative information 5](#_Toc209093066)

[3.1 Study identifiers 5](#_Toc209093067)

[3.2 Revision history 5](#_Toc209093068)

[3.3 Contribution and approvals 5](#_Toc209093069)

[3.3.1 Contributors to the statistical analysis plan 5](#_Toc209093070)

[3.3.2 Approvals 6](#_Toc209093071)

[4 Introduction 7](#_Toc209093072)

[4.1 Study synopsis 7](#_Toc209093073)

[4.2 Study population 7](#_Toc209093074)

[4.2.1 Inclusion criteria 7](#_Toc209093075)

[4.2.2 Exclusion criteria 7](#_Toc209093076)

[4.3 Interventions 8](#_Toc209093077)

[4.4 Study visits 8](#_Toc209093078)

[4.5 Outcomes 9](#_Toc209093079)

[4.5.1 Primary outcome 9](#_Toc209093080)

[4.5.2 Secondary outcomes 9](#_Toc209093081)

[4.5.3 Safety outcomes 9](#_Toc209093082)

[4.6 Randomisation and blinding 9](#_Toc209093083)

[4.7 Sample size 9](#_Toc209093084)

[5 Statistical analysis 9](#_Toc209093085)

[5.1 Statistical hypotheses 9](#_Toc209093086)

[5.2 Statistical principles 10](#_Toc209093087)

[5.2.1 Level of statistical significance 10](#_Toc209093088)

[5.2.2 Statistical software 10](#_Toc209093089)

[5.3 Analysis populations 10](#_Toc209093090)

[5.4 Data description 10](#_Toc209093091)

[5.5 Analysis of the primary outcome 10](#_Toc209093092)

[5.5.1 Unadjusted analysis 10](#_Toc209093093)

[5.5.2 Adjusted analysis 11](#_Toc209093094)

[5.5.3 Missing data handling 11](#_Toc209093095)

[5.5.4 Sensitivity analyses 11](#_Toc209093096)

[5.5.5 Subgroup analyses 11](#_Toc209093097)

[5.6 Analysis of secondary outcomes 12](#_Toc209093098)

[5.7 Adverse Events 12](#_Toc209093099)

[6 References 12](#_Toc209093100)

[7 Proposed outputs 13](#_Toc209093101)

[7.1 Tables 13](#_Toc209093102)

[7.2 Figures 25](#_Toc209093103)

**Tables**

[Table 1. Baseline characteristics of the households 13](#_Toc209092357)

[Table 2 a/b. Baseline characteristics of the participants (a. Adolescents, b. Adults) 13](#_Toc209092358)

[Table 3 a/b. Food intake at baseline (a. Adolescents, b. Adults) 15](#_Toc209092359)

[Table 4. Salt storage at home at baseline 18](#_Toc209092360)

[Table 5 a/b. Compliance (a. Adolescents, b. Adults) 19](#_Toc209092361)

[Table 6. Questionnaire on the study salt at week 16 20](#_Toc209092362)

[Table 7 a/b. Salt knowledge, attitude and behaviour toward dietary salt at week 16 (a. Adolescents, b. Adults) 20](#_Toc209092363)

[Table 8. Spot urine Na/K ratio by visit (a. Adolescents, b. Adults) 22](#_Toc209092364)

[Table 9 a/b. Blood pressure by visit (a. Adolescents, b. Adults) 22](#_Toc209092365)

[Table 10. Model results of the primary and secondary outcomes 23](#_Toc209092366)

**Figures**

[Figure 1. CONSORT diagram 25](#_Toc209092367)

[Figure 2. Longitudinal plot of Na/K+ ratio in Adolescents 26](#_Toc209092368)

[Figure 3. Forest plot for subgroup analysis of change of Na/K ratio at week 16 in adolescents 26](#_Toc209092369)

[Figure 4. Longitudinal plot of Na/K+ ratio in adults 26](#_Toc209092370)

[Figure 5. Longitudinal plot of mean SBP and DBP in adults 26](#_Toc209092371)

[Figure 6. Longitudinal plot of mean SBP and DBP in adolescents 26](#_Toc209092372)

# Administrative information

## Study identifiers

This trial is registered with the Pan African Clinical Trials Registry (<https://pactr.samrc.ac.za>); identifier: PACTR202306727520808

## Revision history

| **Version** | **Date** | **Details** |
| --- | --- | --- |
| 0.1 (draft) | June 2025 | First draft by XL |
| 0.2 (draft) | Aug 2025 | Addressed the comments from SC and LW, change of primary outcome to adolescents only.  Sent to AES and LB for review |
| 1.0 (Final) | Sept 2025 | Addressed the comments from every author, final version |

## Contribution and approvals

### Contributors to the statistical analysis plan

| Name and ORCID | Affiliation | Role on study | SAP contribution |
| --- | --- | --- | --- |
| Xiaoqiu LIU  0000-0001-8620-6592 | The George Institute for Global Health, Faculty of Medicine, University of New South Wales, Sydney, NSW, Australia | Blinded statistician | Developed the initial draft and prepared subsequent versions |
| Simone H CROUCH  0000-0003-1911-5134 | Developmental Pathways for Health Research Unit, University of Witwatersrand | Co-Investigator | Developed the initial draft and reviewed every draft |
| Lisa J WARE  0000-0002-9762-4017 | Developmental Pathways for Health Research Unit, University of Witwatersrand | Co-Investigator | Reviewed every draft |
| Shane A NORRIS  0000-0001-7124-3788 | Developmental Pathways for Health Research Unit, University of Witwatersrand | Co-Investigator | Reviewed every draft |
| Laurent Billot  0000-0002-4975-9793 | The George Institute for Global Health, Faculty of Medicine, University of New South Wales, Sydney, NSW, Australia | Statistician | Reviewed and approved final version |
| Aletta E SCHUTTE 0000-0001-9217-4937 | Professor and Principal Theme Lead of Cardiac, Vascular and Metabolic Medicine  The George Institute for Global Health, University of New South Wales | Principal investigator | Reviewed every draft and approved final version |

### Approvals

The undersigned have reviewed this plan and approve it as final. They find it to be consistent with the requirements of the protocol as it applies to their respective areas. They also find it to be compliant with ICH-E9 principles and, in particular, confirm that this analysis plan was developed in a completely blinded manner (i.e. without knowledge of the effect of the intervention(s) being assessed).

Statistician: Xiaoqiu Liu


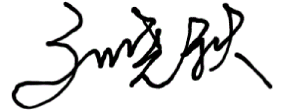
 19 September 2025

Lead Investigator: Aletta E Schutte


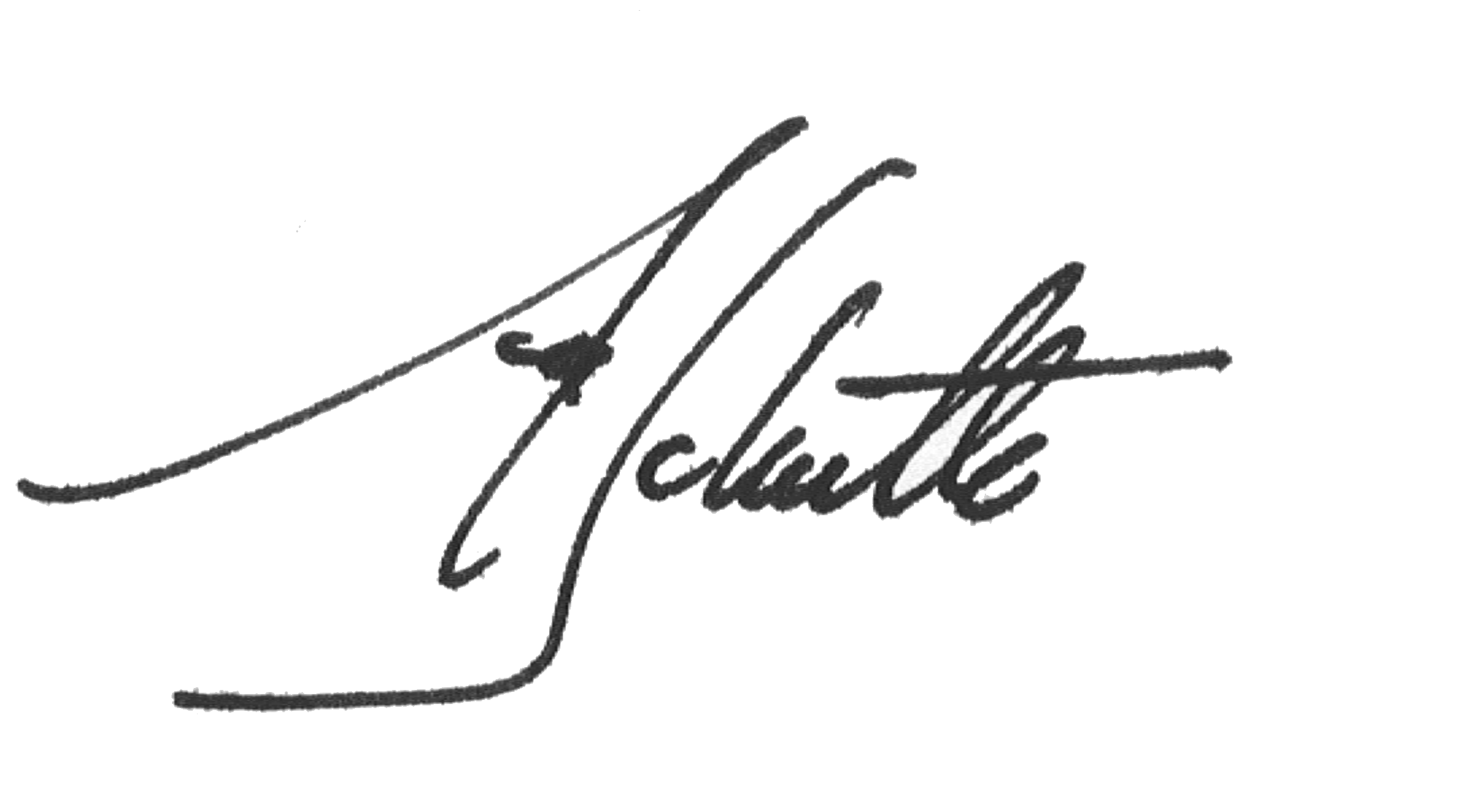
 18 September 2025

# Introduction

## Study synopsis

Sodium in Adolescents and adults Lowering Trial (SALT) study is a phase I, two-arm (1:1) double-blind controlled randomised clinical trial (RCT) on low sodium salt substitute (LSSS). The study includes adolescents, aged 13-19 years, male or female of African descents living in Soweto, South Africa. For each adolescent, one primary caregiver (parent/guardian, aged ≥40 years) was recruited. When the primary caregiver was <40 years of age, an additional member of the household (aged 40-70 years) was included (more details of inclusion and exclusion criteria below in section 4.2). For simplicity, the term “adult” will be used to refer to the primary caregiver and additional members of the household and it does not include adolescents aged 18-19 years.

Adolescents and their households were randomised to receive a LSSS (50% KCl/ 50%NaCl) (intervention, 300 adolescents and 300-600 adults) or traditional table salt (NaCl) (control group, 300 adolescents and 300-600 adults). Both participants and investigators are blinded to the intervention.

This study aims to assess in African families; the effectiveness of a potassium-enriched salt substitute compared to usual salt in improving the urinary sodium-to-potassium ratio and lowering blood pressure (BP) over 4 months.

Patient enrolment was between 08-03-2024 and 30-09-2024.

## Study population

### Inclusion criteria

1. Adolescents, aged 13-19 years, male or female of African descent living in Soweto
2. Adolescent’s primary caregiver (parent/guardian) aged ≥40 years, where the primary caregiver was <40 years of age, an additional member of the household (aged 40-70 years) was included
3. All included household members should eat and stay in the home at least five days a week
4. Individuals presenting with new clinical hypertension were included but referred. Those using BP-lowering medications, specifically angiotensin-converting enzyme inhibitors or Angiotensin receptor blockers, were included

### Exclusion criteria

1. Households where any household member was unable to read or understand English, self-reports any previous diagnosis of or test results showing kidney disease/diminished kidney function, use of potassium-sparing diuretics or potential contraindication to the LSSS, were excluded
2. Households with individuals considered to eat most meals outside of the home, with a previous diagnosis or currently self-reporting any eating disorder, participating in any other trial, high-level athletes, self-reported pregnancy at the time of screening or households without cooking facilities, were ineligible

## Interventions

**Intervention**: low sodium salt substitute, a blend of 50/50 KCl/NaCl manufactured by Cerebos (Cerebos Ltd, South Africa)

**Control**: Traditional table salt, 100% NaCl manufactured by Cerebos

Both groups were asked not to use any other salt products in food within the home. All salt in the household at week zero was removed and weighed, and the equivalent weight of salt was returned to the household at the end of the trial (week 16). Households were contacted regularly to ensure a sufficient supply of salt or salt substitute, and they were additionally given a number to contact should they be running low.

## Study visits

**Table 1.** Schedule of enrolment, interventions, and assessments (adapted from protocol)

| **Measure** | **Completed by** | **Screening** | **Week 0** | **Week 4** | **Week 12** | **Week 16** |
| --- | --- | --- | --- | --- | --- | --- |
| Informed Consent and Assent | All | X |  |  |  |  |
| Test for proteinuria | <18 years | X |  |  |  |  |
| Point-of-care creatinine and eGFR | ≥18 years | X |  |  |  |  |
| Serum creatinine for eGFR | ≥18 years with a point-of-care eGFR 48-72mL/min | X |  |  |  |  |
| Age, gender and education level of all household members | Head of household | X |  |  |  |  |
| Randomisation* | Household |  | X |  |  |  |
| Questionnaires:   - Demographic and health information - Social vulnerability index *(Adapted from Centre for Disease Control social vulnerability index)* - Sleep *(Pittsburgh Sleep Quality Index)* - Physical activity *(Global physical activity questionnaire (GPAQ))* - Food intake *(Healthy and unhealthy food group questionnaire)* | All |  | X |  |  |  |
| Salt storage questionnaire | All |  | X |  |  |  |
| Compliance questionnaire:   - Self-reported used of trial product - Self-reported use of other salt products - Self-reported food consumption outside of the home | All |  |  | X | X | X |
| Salt behaviours questionnaire: Perceived salt use *(Knowledge, Attitudes, Behavior toward Dietary Salt)* | All |  |  |  |  | X |
| Exit question: Participant perceptions of trial | All |  |  |  |  | X |
| Height, Weight and Waist circumference | All |  | X |  |  | X |
| Spot urine to determine Na and K | All |  | X | X | X | X |
| Blood pressure and Heart rate | All |  | X | X | X | X |

Note: * Randomisation was done before Week 0 starts.

An interview was conducted to determine perceptions of the trial relating to the LSSS and the feasibility of its use long-term in Sowetan households by 40 Adolescents total. The interview was conducted in blinded manner.

## Outcomes

### Primary outcome

The primary outcome is the change in spot urinary sodium-to-potassium molar ratio (Na/K ratio) from baseline to 16 weeks in adolescents.

### Secondary outcomes

Secondary outcomes are:

- The change in spot urinary Na/K ratio from baseline to 16 weeks in adults.
- The change in SBP from baseline to 16 weeks in adults
- The change in diastolic BP (DBP) from baseline to 16 weeks in adults.
- The change in SBP from baseline to 16 weeks in adolescents.
- The change in DBP from baseline to 16 weeks in adolescents.

### Safety outcomes

- Any adverse events (AEs) and serious AEs during study period.

## Randomisation and blinding

Adolescents (and their households) were randomised into intervention or control groups using block randomisation to ensure equal group sizes. At randomisation, concealment allocation was strictly observed. All trial staff and researchers were blinded to intervention or control group allocation (double-blind trial design). Trial product was stored in identical unmarked packaging labelled A or B on the bottom of the flask. Unblinded allocation of intervention and control products was known only to an independent team not involved in the trial.

## Sample size

Primary Outcome: A sample size of 470 provides >90% power to detect a 0.6 change in sodium-to-potassium ratio[1, 2] using a 5% significance level (two-sided) assuming a standard deviation of 1.5[3]. Accounting for a 20% dropout, we would recruit a total of 600 adolescents and their primary caregiver.

Secondary Outcome of SBP in adults: A sample size of 488 provides >90% power to detect a 5mmHg difference in the mean systolic BP between the groups using a 5% significant level, assuming a common within group standard deviation (SD) in SBP of 17 mmHg. Where the primary caregiver was younger than 40 years of age, an additional household member aged 40-70 years was recruited, resulting in a total study population of 1200-1800 (600 adolescents and 600-1200 adults).

# Statistical analysis

## Statistical hypotheses

The primary statistical hypotheses are as follows:

- **Null hypothesis**: The intervention, potassium-enriched salt substitute, has similar effect as traditional table salt on spot urinary Na/K ratio in people aged 13-19 years.
- **Alternative hypothesis (2-sided)**: The intervention, potassium-enriched salt substitute is better than traditional table salt in lowering the spot urinary Na/K ratio (closer to 1) from baseline to 16 weeks in adolescents.

## Statistical principles

### Level of statistical significance

No interim analysis was previously conducted. Thus, the significance threshold will remain at 5% for the final analysis. All tests are to be two-sided with a nominal level of α set at 5%.

Analyses of the primary outcome (spot urinary Na/K ratio) will be unadjusted for multiplicity; however, the family-wise error rate will be controlled across secondary outcomes (one family) outcomes (one family) using a Holm-Bonferroni correction[4]. No other multiplicity adjustment will be applied.

### Statistical software

Analyses will be conducted primarily using SAS (version 9.3 or above) or R (version R 4.3.1 or above).

## Analysis populations

Intention-to-treat (ITT) analysis set: All randomised participants will be analysed according to their randomised group, regardless of adherence to the protocol, and excluding those who withdrew their consent. This will be the primary analysis set to assess both effectiveness and safety. The flow of participants through the study will be displayed in a CONSORT diagram (Figure 1).

Per Protocol (PP) analysis set: All participants in ITT dataset without major protocol deviation. The major protocol deviations are: Household that used study salt for less than 20% of the meals cooked at home; participants that have eaten out more than half of the meals. The PP set will be used to rerun analyses of the primary outcome, including sensitivity analyses. Subgroup analysis will not be performed in PP set.

## Data description

Baseline characteristics and all the outcome variables will be presented by randomisation group, in adolescents and adult participants, separately (except for the household variables). Discrete variables will be summarised by frequencies and percentages. Percentages will be calculated in patients whose data are available. Continuous variables will be summarised by using mean and SD, and median and interquartile range (Q1-Q3).

## Analysis of the primary outcome

Analyses of the primary outcome will be run in both the ITT and PP populations.

### Unadjusted analysis

The primary outcome will be analysed using repeated measure generalized linear mixed model. The model will include outcome data collected at baseline and every follow-up visit. Fixed effects will include the randomised treatment allocation, the visit as a categorical variable with 4 levels (i.e. baseline, weeks 4, 12 and 16), the interaction between treatment and visit. Each individual participant will be included as a random effect, and the within-participant correlation between repeated measures will be modelled using compound-symmetry structure. The intervention effect will be estimated as the difference of the change in Na/K ratio (Difference in differences, DiD) from baseline to week 16 and its 95% Confidence interval (CI) between the two groups (Intervention minus Control).

### Adjusted analysis

Adjusted analysis will be performed by adding the adjustment for age and sex to the main model. A blinded data review will be conducted to check if any other factors to be included.

The adjusted analysis will be the primary analysis for the primary outcome.

### Missing data handling

Missing data will be examined for the analysis of the primary outcome. We will report the percentage of adolescent participants missing Na/K ratio at baseline and each follow-up visit. We will also report the number of participants included in the primary analysis, i.e., having all the covariates (e.g., age, sex) and at least one of the Na/K ratios at baseline and follow-up visits). If the proportion of participants excluded from the primary analysis is <= 5% of all the randomised patients, no multiple imputation (MI) is need. Otherwise, we will perform MI assuming missing at random (MAR).

The imputation model will include the treatment arm, all baseline variables (listed in Table 2) and available Na/K ratio at baseline and follow-up visits. One hundred sets will be imputed using fully conditional specification with discrete variables imputed using a discriminant function[5]. The imputed data will be analysed using the same model as the one used for the main analysis, i.e. the adjusted model.

### Sensitivity analyses

If the missing data from the primary analysis is <= 5%, the adjusted model results using available data will remain the primary analysis. If missing is greater than 5%, the adjusted model results using MI data will be considered as the primary analysis.

### Subgroup analyses

Potential interactions will be explored for spot urinary Na/K ratio and SBP in both adolescents and adults using the following baseline variables and tested at the p=0.05 level. The subgroup analyses are exploratory and will be carried out irrespective of whether there is a significant treatment effect on the primary outcome.

Subgroups in adolescents:

- Sex (Female/Male)
- Hypertension (Yes/No)
- Body mass index (Overweight (>+1SD, equivalent to BMI 25 kg/m2 at 19 years)/ Obesity (>+2SD, equivalent to BMI 30 kg/m2 at 19 years) / Thinness: <-2SD /Severe thinness: <-3SD)[6]

Subgroups in adults:

- Age groups (20-40/ >40)
- Sex (Female/Male)
- Hypertension (Yes/No)
- Body Mass index (≤18.5, 18.51-24.99, 25-29.99, ≥30 kg/m^2^)

The analysis for each subgroup will be performed by adding the subgroup variable, as well as its interaction with the intervention as a fixed effect to the main mixed model. Collinearity should be avoided when adding the subgroup variable in, e.g., when age group is added, the age as continuous variable in the main model should be removed. Within each subgroup, summary measures will include raw numbers and percentages of the participants within each treatment arm, as well as the DiD for treatment effect with a 95% CI. The results will be displayed in a forest plot including the P values for heterogeneity corresponding to the interaction term between the intervention and the subgroup variables. Given the main purpose of these subgroup analyses is exploratory and to assess the consistency of results, they will only be performed on the non-imputed data (i.e. using all data available).

## Analysis of secondary outcomes

The adjusted model described in section 5.5.2 will be used for the analysis of the secondary outcomes based on the available data (non-imputation is needed).

## Adverse Events

The blinded review indicated that only 4 Adverse events (AEs) occurred in this study, it will be presented descriptively (AE term, onset time, allocation group, severity, AE outcome, relationship to the study salt).

# References

1. Groenland, E.H., et al., *Validation of spot urine in estimating 24-h urinary sodium, potassium and sodium-to-potassium ratio during three different sodium diets in healthy adults.* Blood Press, 2023. **32**(1): p. 2170868.

2. Yatabe, M.S., et al., *Urinary Sodium-to-Potassium Ratio Tracks the Changes in Salt Intake during an Experimental Feeding Study Using Standardized Low-Salt and High-Salt Meals among Healthy Japanese Volunteers.* Nutrients, 2017. **9**(9).

3. Carla El Mallah, K.M., Hala Ghattas, Dareen Shatila, Sirine Francis, Sani Hlais, Imad Toufeili & Omar Obeid, *Elevated urinary Na/K ratio among Lebanese elementary school children is attributable to low K intake.* European Journal of Nutrition, 2017. **56**: p. 1149-1156.

4. Holm, S., *A Simple Sequentially Rejective Multiple Test Procedure.* Scandinavian Journal of Statistics, 1979. **6**(2): p. 65-70.

5. van Buuren, S., *Multiple imputation of discrete and continuous data by fully conditional specification.* Stat Methods Med Res, 2007. **16**(3): p. 219-42.

6. WHO, *Growth reference data for 5-19 years* [*https://www.who.int/tools/growth-reference-data-for-5to19-years/indicators/bmi-for-age*](https://www.who.int/tools/growth-reference-data-for-5to19-years/indicators/bmi-for-age). 2025.

# Proposed outputs

## Tables

Table 1. Baseline characteristics of the households

| Household | Potassium-enriched salt substitute  (N = ) | Traditional table salt  (N = ) | Total  (N = ) |
| --- | --- | --- | --- |
| Residents per household | xxx | xxx | xxx |
| Mean (SD) | xxx.x (xxx.x) | xxx.x (xxx.x) | xxx.x (xxx.x) |
| Median (Q1; Q3) | xxx (xxx; xxx) | xxx (xxx; xxx) | xxx (xxx; xxx) |
| min max | xxx to xxx | xxx to xxx | xxx to xxx |
|  |  |  |  |
| Age (years) | xxx | xxx | xxx |
| Mean (SD) | xxx.x (xxx.x) | xxx.x (xxx.x) | xxx.x (xxx.x) |
| Median (Q1; Q3) | xxx (xxx; xxx) | xxx (xxx; xxx) | xxx (xxx; xxx) |
| min max | xxx to xxx | xxx to xxx | xxx to xxx |

Table 2 a/b. Baseline characteristics of the participants (a. Adolescents, b. Adults)

| Participants | Potassium-enriched salt substitute  (N = ) | Traditional table salt  (N = ) | Total  (N = ) |
| --- | --- | --- | --- |
| Age (years) | xxx | xxx | xxx |
| Mean (SD) | xxx.x (xxx.x) | xxx.x (xxx.x) | xxx.x (xxx.x) |
| Median (Q1; Q3) | xxx (xxx; xxx) | xxx (xxx; xxx) | xxx (xxx; xxx) |
| min max | xxx to xxx | xxx to xxx | xxx to xxx |
|  |  |  |  |
| Sex | xxx | xxx | xxx |
| Male | xxx xx.x% | xxx xx.x% | xxx xx.x% |
| Female | xxx xx.x% | xxx xx.x% | xxx xx.x% |
| Other |  |  |  |
|  |  |  |  |
| Country of birth | xxx | xxx | xxx |
| South Africa | xxx xx.x% | xxx xx.x% | xxx xx.x% |
| Other | xxx xx.x% | xxx xx.x% | xxx xx.x% |
|  |  |  |  |
| Anthropometrics |  |  |  |
| Height (cm) | xxx | xxx | xxx |
| Mean (SD) | xxx.x (xxx.x) | xxx.x (xxx.x) | xxx.x (xxx.x) |
| Median (Q1; Q3) | xxx (xxx; xxx) | xxx (xxx; xxx) | xxx (xxx; xxx) |
| min max | xxx to xxx | xxx to xxx | xxx to xxx |
|  |  |  |  |
| Weight (kg) | xxx | xxx | xxx |
| Mean (SD) | xxx.x (xxx.x) | xxx.x (xxx.x) | xxx.x (xxx.x) |
| Median (Q1; Q3) | xxx (xxx; xxx) | xxx (xxx; xxx) | xxx (xxx; xxx) |
| min max | xxx to xxx | xxx to xxx | xxx to xxx |
|  |  |  |  |
| BMI (kg/m2) | xxx | xxx | xxx |
| Mean (SD) | xxx.x (xxx.x) | xxx.x (xxx.x) | xxx.x (xxx.x) |
| Median (Q1; Q3) | xxx (xxx; xxx) | xxx (xxx; xxx) | xxx (xxx; xxx) |
| min max | xxx to xxx | xxx to xxx | xxx to xxx |
|  |  |  |  |
| Waist circumference (cm) | xxx | xxx | xxx |
| Mean (SD) | xxx.x (xxx.x) | xxx.x (xxx.x) | xxx.x (xxx.x) |
| Median (Q1; Q3) | xxx (xxx; xxx) | xxx (xxx; xxx) | xxx (xxx; xxx) |
| min max | xxx to xxx | xxx to xxx | xxx to xxx |
|  |  |  |  |
| Years of education | xxx | xxx | xxx |
| Mean (SD) | xxx.x (xxx.x) | xxx.x (xxx.x) | xxx.x (xxx.x) |
| Median (Q1; Q3) | xxx (xxx; xxx) | xxx (xxx; xxx) | xxx (xxx; xxx) |
| min max | xxx to xxx | xxx to xxx | xxx to xxx |
|  |  |  |  |
| Highest degree | xxx | xxx | xxx |
| Grade 12 | xxx xx.x% | xxx xx.x% | xxx xx.x% |
| Diploma | xxx xx.x% | xxx xx.x% | xxx xx.x% |
| Bachelor and above | xxx xx.x% | xxx xx.x% | xxx xx.x% |
|  |  |  |  |
| Occupation | xxx | xxx | xxx |
| Student | xxx xx.x% | xxx xx.x% | xxx xx.x% |
| Employed/ Self Employed | xxx xx.x% | xxx xx.x% | xxx xx.x% |
| Unemployed | xxx xx.x% | xxx xx.x% | xxx xx.x% |
| Retired | xxx xx.x% | xxx xx.x% | xxx xx.x% |
|  |  |  |  |
| Medical History |  |  |  |
| Hypertension* | xxx | xxx | xxx |
| Taking hypertension medication | xxx xx.x% | xxx xx.x% | xxx xx.x% |
| Controlled on treatment | xxx xx.x% | xxx xx.x% | xxx xx.x% |
| High cholesterol | xxx xx.x% | xxx xx.x% | xxx xx.x% |
| Taking cholesterol lowing medication | xxx xx.x% | xxx xx.x% | xxx xx.x% |
| Tuberculosis | xxx xx.x% | xxx xx.x% | xxx xx.x% |
| Taking medication for tuberculosis | xxx xx.x% | xxx xx.x% | xxx xx.x% |
| Depression | xxx xx.x% | xxx xx.x% | xxx xx.x% |
| Taking medication for depression | xxx xx.x% | xxx xx.x% | xxx xx.x% |
| Diabetes | xxx xx.x% | xxx xx.x% | xxx xx.x% |
| Taking medication for diabetes | xxx xx.x% | xxx xx.x% | xxx xx.x% |
| HIV | xxx xx.x% | xxx xx.x% | xxx xx.x% |
| Taking antiretroviral medication | xxx xx.x% | xxx xx.x% | xxx xx.x% |
|  |  |  |  |
| Modifiable Risk Factor |  |  |  |
| Cigarette Smoking | xxx | xxx | xxx |
| Never | xxx xx.x% | xxx xx.x% | xxx xx.x% |
| Former smoker | xxx xx.x% | xxx xx.x% | xxx xx.x% |
| Current smoker | xxx xx.x% | xxx xx.x% | xxx xx.x% |
|  |  |  |  |
| Alcohol Drinking | xxx | xxx | xxx |
| Low-risk consumption | xxx xx.x% | xxx xx.x% | xxx xx.x% |
| Hazardous or harmful consumption | xxx xx.x% | xxx xx.x% | xxx xx.x% |
| Alcohol dependence | xxx xx.x% | xxx xx.x% | xxx xx.x% |
|  |  |  |  |
| Recreational Drug use | xxx xx.x% | xxx xx.x% | xxx xx.x% |
|  |  |  |  |
| Physical Activity |  |  |  |
| Moderate to Vigorous Physical | xxx | xxx | xxx |
| Mean (SD) | xxx.x (xxx.x) | xxx.x (xxx.x) | xxx.x (xxx.x) |
| Median (Q1; Q3) | xxx.x (xxx.x) | xxx.x (xxx.x) | xxx.x (xxx.x) |
| min max | xxx to xxx | xxx to xxx | xxx to xxx |
| ≥ 150 minutes per week | xxx xx.x% | xxx xx.x% | xxx xx.x% |
| <150 minutes per week | xxx xx.x% | xxx xx.x% | xxx xx.x% |
|  |  |  |  |
| Global Pittsburgh Sleep Quality Index |  |  |  |
| Mean (SD) | xxx.x (xxx.x) | xxx.x (xxx.x) | xxx.x (xxx.x) |
| Median (Q1; Q3) | xxx.x (xxx.x) | xxx.x (xxx.x) | xxx.x (xxx.x) |
| min max | xxx to xxx | xxx to xxx | xxx to xxx |

Note: * Hypertension data will be from the survey and the actual baseline blood pressure measurement.

Table 3 a/b. Food intake at baseline (a. Adolescents, b. Adults)

|  | Potassium-enriched salt substitute  (N = ) | Traditional table salt  (N = ) | Total  (N = ) |
| --- | --- | --- | --- |
| Fruits (excluding canned fruits) | xxx | xxx | xxx |
| 0 days | xxx xx.x% | xxx xx.x% | xxx xx.x% |
| 1-2 days | xxx xx.x% | xxx xx.x% | xxx xx.x% |
| 3-4 days | xxx xx.x% | xxx xx.x% | xxx xx.x% |
| 5-6 days | xxx xx.x% | xxx xx.x% | xxx xx.x% |
| 7 days | xxx xx.x% | xxx xx.x% | xxx xx.x% |
|  |  |  |  |
| Vegetables | xxx | xxx | xxx |
| 0 days | xxx xx.x% | xxx xx.x% | xxx xx.x% |
| 1-2 days | xxx xx.x% | xxx xx.x% | xxx xx.x% |
| 3-4 days | xxx xx.x% | xxx xx.x% | xxx xx.x% |
| 5-6 days | xxx xx.x% | xxx xx.x% | xxx xx.x% |
| 7 days | xxx xx.x% | xxx xx.x% | xxx xx.x% |
|  |  |  |  |
| Meat, chicken, eggs or fish | xxx | xxx | xxx |
| 0 days | xxx xx.x% | xxx xx.x% | xxx xx.x% |
| 1-2 days | xxx xx.x% | xxx xx.x% | xxx xx.x% |
| 3-4 days | xxx xx.x% | xxx xx.x% | xxx xx.x% |
| 5-6 days | xxx xx.x% | xxx xx.x% | xxx xx.x% |
| 7 days | xxx xx.x% | xxx xx.x% | xxx xx.x% |
|  |  |  |  |
| Milk or maas (inkhomazi, mothembi) | xxx | xxx | xxx |
| 0 days | xxx xx.x% | xxx xx.x% | xxx xx.x% |
| 1-2 days | xxx xx.x% | xxx xx.x% | xxx xx.x% |
| 3-4 days | xxx xx.x% | xxx xx.x% | xxx xx.x% |
| 5-6 days | xxx xx.x% | xxx xx.x% | xxx xx.x% |
| 7 days | xxx xx.x% | xxx xx.x% | xxx xx.x% |
|  |  |  |  |
| Cold drinks (fizzy drinks or cordials) | xxx | xxx | xxx |
| 0 days | xxx xx.x% | xxx xx.x% | xxx xx.x% |
| 1-2 days | xxx xx.x% | xxx xx.x% | xxx xx.x% |
| 3-4 days | xxx xx.x% | xxx xx.x% | xxx xx.x% |
| 5-6 days | xxx xx.x% | xxx xx.x% | xxx xx.x% |
| 7 days | xxx xx.x% | xxx xx.x% | xxx xx.x% |
|  |  |  |  |
| Sugar in tea (teaspoons sugar/cup) | xxx | xxx | xxx |
| 0 days | xxx xx.x% | xxx xx.x% | xxx xx.x% |
| 1-2 days | xxx xx.x% | xxx xx.x% | xxx xx.x% |
| 3-4 days | xxx xx.x% | xxx xx.x% | xxx xx.x% |
| 5-6 days | xxx xx.x% | xxx xx.x% | xxx xx.x% |
| 7 days | xxx xx.x% | xxx xx.x% | xxx xx.x% |
|  |  |  |  |
| Cookies or cake | xxx | xxx | xxx |
| 0 days | xxx xx.x% | xxx xx.x% | xxx xx.x% |
| 1-2 days | xxx xx.x% | xxx xx.x% | xxx xx.x% |
| 3-4 days | xxx xx.x% | xxx xx.x% | xxx xx.x% |
| 5-6 days | xxx xx.x% | xxx xx.x% | xxx xx.x% |
| 7 days | xxx xx.x% | xxx xx.x% | xxx xx.x% |
|  |  |  |  |
| Chips or cheese puffs or niknaks | xxx | xxx | xxx |
| 0 days | xxx xx.x% | xxx xx.x% | xxx xx.x% |
| 1-2 days | xxx xx.x% | xxx xx.x% | xxx xx.x% |
| 3-4 days | xxx xx.x% | xxx xx.x% | xxx xx.x% |
| 5-6 days | xxx xx.x% | xxx xx.x% | xxx xx.x% |
| 7 days | xxx xx.x% | xxx xx.x% | xxx xx.x% |
|  |  |  |  |
| Sweets or chocolates | xxx | xxx | xxx |
| 0 days | xxx xx.x% | xxx xx.x% | xxx xx.x% |
| 1-2 days | xxx xx.x% | xxx xx.x% | xxx xx.x% |
| 3-4 days | xxx xx.x% | xxx xx.x% | xxx xx.x% |
| 5-6 days | xxx xx.x% | xxx xx.x% | xxx xx.x% |
| 7 days | xxx xx.x% | xxx xx.x% | xxx xx.x% |
|  |  |  |  |
| Fast food (Fried chicken, hamburger, chips, pie, pizza, polony) | xxx | xxx | xxx |
| 0 days | xxx xx.x% | xxx xx.x% | xxx xx.x% |
| 1-2 days | xxx xx.x% | xxx xx.x% | xxx xx.x% |
| 3-4 days | xxx xx.x% | xxx xx.x% | xxx xx.x% |
| 5-6 days | xxx xx.x% | xxx xx.x% | xxx xx.x% |
| 7 days | xxx xx.x% | xxx xx.x% | xxx xx.x% |
|  |  |  |  |
| Bread | xxx | xxx | xxx |
| 0 days | xxx xx.x% | xxx xx.x% | xxx xx.x% |
| 1-2 days | xxx xx.x% | xxx xx.x% | xxx xx.x% |
| 3-4 days | xxx xx.x% | xxx xx.x% | xxx xx.x% |
| 5-6 days | xxx xx.x% | xxx xx.x% | xxx xx.x% |
| 7 days | xxx xx.x% | xxx xx.x% | xxx xx.x% |
|  |  |  |  |

Table 4. Salt storage at home at baseline

|  | Potassium-enriched salt substitute  (N = ) | Traditional table salt  (N = ) | Total  (N = ) |
| --- | --- | --- | --- |
| Where in the house is salt stored? | xxx | xxx | xxx |
| Kitchen cupboard | xxx xx.x% | xxx xx.x% | xxx xx.x% |
| Kitchen counter | xxx xx.x% | xxx xx.x% | xxx xx.x% |
| On the table | xxx xx.x% | xxx xx.x% | xxx xx.x% |
| Alternative eating area | xxx xx.x% | xxx xx.x% | xxx xx.x% |
| Other | xxx xx.x% | xxx xx.x% | xxx xx.x% |
|  |  |  |  |
| How is salt stored in the house? | xxx | xxx | xxx |
| In plastic package | xxx xx.x% | xxx xx.x% | xxx xx.x% |
| In a saltshaker | xxx xx.x% | xxx xx.x% | xxx xx.x% |
| In a manufactures salt dispenser (large) | xxx xx.x% | xxx xx.x% | xxx xx.x% |
| In a manufactures salt dispenser (small) | xxx xx.x% | xxx xx.x% | xxx xx.x% |
| Other | xxx xx.x% | xxx xx.x% | xxx xx.x% |
|  |  |  |  |
| What salt products are in the house? | xxx | xxx | xxx |
| Regular salt (NaCl) | xxx xx.x% | xxx xx.x% | xxx xx.x% |
| Himalayan/ pink salt | xxx xx.x% | xxx xx.x% | xxx xx.x% |
| Salt alternative/ substitute | xxx xx.x% | xxx xx.x% | xxx xx.x% |
|  |  |  |  |
| What additional high salt products are in the house? | xxx | xxx | xxx |
| Stock cubes/powder | xxx xx.x% | xxx xx.x% | xxx xx.x% |
| Soup powder | xxx xx.x% | xxx xx.x% | xxx xx.x% |
| Aromat | xxx xx.x% | xxx xx.x% | xxx xx.x% |
| Bisto Gravy Powder | xxx xx.x% | xxx xx.x% | xxx xx.x% |
| Other | xxx xx.x% | xxx xx.x% | xxx xx.x% |

Table 5 a/b. Compliance (a. Adolescents, b. Adults)

|  | Potassium-enriched salt substitute  (N = ) | Traditional table salt  (N = ) | Total  (N = ) |
| --- | --- | --- | --- |
| How many days in last week did you use the study salt provided? | xxx | xxx | xxx |
| Mean (SD) | xxx.x (xxx.x) | xxx.x (xxx.x) | xxx.x (xxx.x) |
| Median (Q1; Q3) | xxx (xxx; xxx) | xxx (xxx; xxx) | xxx (xxx; xxx) |
| min max | xxx to xxx | xxx to xxx | xxx to xxx |
|  |  |  |  |
| For how many meals did you use the study salt during a typical day? | xxx | xxx | xxx |
| Only once | xxx xx.x% | xxx xx.x% | xxx xx.x% |
| Half of the meals | xxx xx.x% | xxx xx.x% | xxx xx.x% |
| All means only | xxx xx.x% | xxx xx.x% | xxx xx.x% |
| All meals and snacks | xxx xx.x% | xxx xx.x% | xxx xx.x% |
|  |  |  |  |
| How did you use the study salt?* | xxx | xxx | xxx |
| During cooking | xxx xx.x% | xxx xx.x% | xxx xx.x% |
| As seasoning on already cooked food | xxx xx.x% | xxx xx.x% | xxx xx.x% |
| Did not use | xxx xx.x% | xxx xx.x% | xxx xx.x% |
|  |  |  |  |
| Have you used any other salt used since the last visit? |  |  |  |
| Yes | xxx xx.x% | xxx xx.x% | xxx xx.x% |
| No | xxx xx.x% | xxx xx.x% | xxx xx.x% |
|  |  |  |  |
| Have you eaten any meals outside of the home? |  |  |  |
| Yes | xxx xx.x% | xxx xx.x% | xxx xx.x% |
| No | xxx xx.x% | xxx xx.x% | xxx xx.x% |

Note: *the first two answers are not mutually exclusive

Compliance information is collected at week 4, 12 and 16. The statistical report will show the raw descriptive data per week.

Table 6. Questionnaire on the study salt at week 16

|  | Potassium-enriched salt substitute  (N = ) | Traditional table salt  (N = ) | Total  (N = ) |
| --- | --- | --- | --- |
| Which allocated group the participants thought they were in | xxx | xxx | xxx |
| Potassium enriched salt substitute | xxx xx.x% | xxx xx.x% | xxx xx.x% |
| Traditional table salt | xxx xx.x% | xxx xx.x% | xxx xx.x% |
|  |  |  |  |
| The reason of perception | xxx | xxx | xxx |
| The salt tasted different to regular salt | xxx xx.x% | xxx xx.x% | xxx xx.x% |
| The salt looked different to regular salt | xxx xx.x% | xxx xx.x% | xxx xx.x% |
| I feel different | xxx xx.x% | xxx xx.x% | xxx xx.x% |
| Other | xxx xx.x% | xxx xx.x% | xxx xx.x% |
|  |  |  |  |
| Happy to use this salt substitute? | xxx | xxx | xxx |
| Yes | xxx xx.x% | xxx xx.x% | xxx xx.x% |
| No | xxx xx.x% | xxx xx.x% | xxx xx.x% |
|  |  |  |  |
| If not, why not? | xxx | xxx | xxx |
| I did not like the taste | xxx xx.x% | xxx xx.x% | xxx xx.x% |
| I am worried it may cost more | xxx xx.x% | xxx xx.x% | xxx xx.x% |
| I don’t think it has any benefit | xxx xx.x% | xxx xx.x% | xxx xx.x% |
| Other | xxx xx.x% | xxx xx.x% | xxx xx.x% |

Table 7 a/b. Salt knowledge, attitude and behaviour toward dietary salt at week 16 (a. Adolescents, b. Adults)

|  | Potassium-enriched salt substitute  (N = ) | Traditional table salt  (N = ) | Total  (N = ) |
| --- | --- | --- | --- |
| Study salt used per person* | xxx | xxx | xxx |
| Mean (SD) | xxx.x (xxx.x) | xxx.x (xxx.x) | xxx.x (xxx.x) |
| Median (Q1; Q3) | xxx (xxx; xxx) | xxx (xxx; xxx) | xxx (xxx; xxx) |
| min max | xxx to xxx | xxx to xxx | xxx to xxx |
|  |  |  |  |
| Do you add salt to food at the table? | xxx | xxx | xxx |
| Never | xxx xx.x% | xxx xx.x% | xxx xx.x% |
| Rarely | xxx xx.x% | xxx xx.x% | xxx xx.x% |
| Sometimes | xxx xx.x% | xxx xx.x% | xxx xx.x% |
| Often | xxx xx.x% | xxx xx.x% | xxx xx.x% |
| Always | xxx xx.x% | xxx xx.x% | xxx xx.x% |
|  |  |  |  |
| In the food you eat at home: is salt added during cooking? | xxx | xxx | xxx |
| Never | xxx xx.x% | xxx xx.x% | xxx xx.x% |
| Rarely | xxx xx.x% | xxx xx.x% | xxx xx.x% |
| Sometimes | xxx xx.x% | xxx xx.x% | xxx xx.x% |
| Often | xxx xx.x% | xxx xx.x% | xxx xx.x% |
| Always | xxx xx.x% | xxx xx.x% | xxx xx.x% |
|  |  |  |  |
| How much salt do you think you consume? | xxx | xxx | xxx |
| Far too much | xxx xx.x% | xxx xx.x% | xxx xx.x% |
| Too much | xxx xx.x% | xxx xx.x% | xxx xx.x% |
| Just the right amount | xxx xx.x% | xxx xx.x% | xxx xx.x% |
| Too little | xxx xx.x% | xxx xx.x% | xxx xx.x% |
| Far too little | xxx xx.x% | xxx xx.x% | xxx xx.x% |
| Refused | xxx xx.x% | xxx xx.x% | xxx xx.x% |
| Don’t know | xxx xx.x% | xxx xx.x% | xxx xx.x% |
|  |  |  |  |
| Do you think that a high salt diet could cause a serious health problem? | xxx | xxx | xxx |
| Yes | xxx xx.x% | xxx xx.x% | xxx xx.x% |
| No | xxx xx.x% | xxx xx.x% | xxx xx.x% |
| Refused | xxx xx.x% | xxx xx.x% | xxx xx.x% |
| Don’t know | xxx xx.x% | xxx xx.x% | xxx xx.x% |
|  |  |  |  |
| How important to you is lowering the salt/sodium in your diet? | xxx | xxx | xxx |
| Not at all | xxx xx.x% | xxx xx.x% | xxx xx.x% |
| Somewhat | xxx xx.x% | xxx xx.x% | xxx xx.x% |
| Very important | xxx xx.x% | xxx xx.x% | xxx xx.x% |
|  |  |  |  |
| Do you do anything on a regular basis to control your salt or sodium intake? | xxx | xxx | xxx |
| Yes | xxx xx.x% | xxx xx.x% | xxx xx.x% |
| No | xxx xx.x% | xxx xx.x% | xxx xx.x% |
| Refused | xxx xx.x% | xxx xx.x% | xxx xx.x% |
| Don’t know | xxx xx.x% | xxx xx.x% | xxx xx.x% |
| If yes: |  |  |  |
| Try to eat less processed foods (like bread, crispy chips, sauces, soups) | xxx xx.x% | xxx xx.x% | xxx xx.x% |
| Look at the salt or sodium labels on food | xxx xx.x% | xxx xx.x% | xxx xx.x% |
| Do not add salt at the table | xxx xx.x% | xxx xx.x% | xxx xx.x% |
| Buy low salt/sodium alternatives | xxx xx.x% | xxx xx.x% | xxx xx.x% |
| Do not add salt when cooking | xxx xx.x% | xxx xx.x% | xxx xx.x% |
| Use spices other than salt when cooking | xxx xx.x% | xxx xx.x% | xxx xx.x% |
| Avoid eating out | xxx xx.x% | xxx xx.x% | xxx xx.x% |
| Other | xxx xx.x% | xxx xx.x% | xxx xx.x% |

Programming note:

* Should be calculated from salt distribution and re-collection. Averaged from household usage.

Table 8. Spot urine Na/K ratio by visit (a. Adolescents, b. Adults)

| Na/K ratio in adults | Potassium-enriched salt substitute  (N = ) | Traditional table salt  (N = ) | Total  (N = ) |
| --- | --- | --- | --- |
| Baseline |  |  |  |
| Mean (SD) | xxx.x (xxx.x) | xxx.x (xxx.x) | xxx.x (xxx.x) |
| Median (Q1; Q3) | xxx (xxx; xxx) | xxx (xxx; xxx) | xxx (xxx; xxx) |
| min max | xxx to xxx | xxx to xxx | xxx to xxx |
| Week 4 |  |  |  |
| Mean (SD) | xxx.x (xxx.x) | xxx.x (xxx.x) | xxx.x (xxx.x) |
| Median (Q1; Q3) | xxx (xxx; xxx) | xxx (xxx; xxx) | xxx (xxx; xxx) |
| min max | xxx to xxx | xxx to xxx | xxx to xxx |
| Week 12 |  |  |  |
| Mean (SD) | xxx.x (xxx.x) | xxx.x (xxx.x) | xxx.x (xxx.x) |
| Median (Q1; Q3) | xxx (xxx; xxx) | xxx (xxx; xxx) | xxx (xxx; xxx) |
| min max | xxx to xxx | xxx to xxx | xxx to xxx |
| Week 16 |  |  |  |
| Mean (SD) | xxx.x (xxx.x) | xxx.x (xxx.x) | xxx.x (xxx.x) |
| Median (Q1; Q3) | xxx (xxx; xxx) | xxx (xxx; xxx) | xxx (xxx; xxx) |
| min max | xxx to xxx | xxx to xxx | xxx to xxx |

Table 9 a/b. Blood pressure by visit (a. Adolescents, b. Adults)

|  | Potassium-enriched salt substitute  (N = ) | Traditional table salt  (N = ) | Total  (N = ) |
| --- | --- | --- | --- |
| SBP |  |  |  |
| Baseline |  |  |  |
| Mean (SD) | xxx.x (xxx.x) | xxx.x (xxx.x) | xxx.x (xxx.x) |
| Median (Q1; Q3) | xxx (xxx; xxx) | xxx (xxx; xxx) | xxx (xxx; xxx) |
| min max | xxx to xxx | xxx to xxx | xxx to xxx |
| Week 4 |  |  |  |
| Mean (SD) | xxx.x (xxx.x) | xxx.x (xxx.x) | xxx.x (xxx.x) |
| Median (Q1; Q3) | xxx (xxx; xxx) | xxx (xxx; xxx) | xxx (xxx; xxx) |
| min max | xxx to xxx | xxx to xxx | xxx to xxx |
| Week 12 |  |  |  |
| Mean (SD) | xxx.x (xxx.x) | xxx.x (xxx.x) | xxx.x (xxx.x) |
| Median (Q1; Q3) | xxx (xxx; xxx) | xxx (xxx; xxx) | xxx (xxx; xxx) |
| min max | xxx to xxx | xxx to xxx | xxx to xxx |
| Week 16 | xxx | xxx | xxx |
| Mean (SD) | xxx.x (xxx.x) | xxx.x (xxx.x) | xxx.x (xxx.x) |
| Median (Q1; Q3) | xxx (xxx; xxx) | xxx (xxx; xxx) | xxx (xxx; xxx) |
| min max | xxx to xxx | xxx to xxx | xxx to xxx |
|  |  |  |  |
| DBP | xxx | xxx | xxx |
| Baseline |  |  |  |
| Mean (SD) | xxx.x (xxx.x) | xxx.x (xxx.x) | xxx.x (xxx.x) |
| Median (Q1; Q3) | xxx (xxx; xxx) | xxx (xxx; xxx) | xxx (xxx; xxx) |
| min max | xxx to xxx | xxx to xxx | xxx to xxx |
| Week 4 |  |  |  |
| Mean (SD) | xxx.x (xxx.x) | xxx.x (xxx.x) | xxx.x (xxx.x) |
| Median (Q1; Q3) | xxx (xxx; xxx) | xxx (xxx; xxx) | xxx (xxx; xxx) |
| min max | xxx to xxx | xxx to xxx | xxx to xxx |
| Week 12 | xxx | xxx | xxx |
| Mean (SD) | xxx.x (xxx.x) | xxx.x (xxx.x) | xxx.x (xxx.x) |
| Median (Q1; Q3) | xxx (xxx; xxx) | xxx (xxx; xxx) | xxx (xxx; xxx) |
| min max | xxx to xxx | xxx to xxx | xxx to xxx |
| Week 16 | xxx | xxx | xxx |
| Mean (SD) | xxx.x (xxx.x) | xxx.x (xxx.x) | xxx.x (xxx.x) |
| Median (Q1; Q3) | xxx (xxx; xxx) | xxx (xxx; xxx) | xxx (xxx; xxx) |
| min max | xxx to xxx | xxx to xxx | xxx to xxx |

Table 10. Model results of the primary and secondary outcomes

| Outcome and Model | Unadjusted Model | Adjusted model | | |
| --- | --- | --- | --- | --- |
|  | DiD (95% CI) | DiD (95% CI) | p | Multiple adjusted p* |
| Primary outcome |  |  |  |  |
| Na/K ratio at week 16 in adolescents | xx.xx (xx.xx to xx.xx) | xx.xx (xx.xx to xx.xx) | 0.xxx | - |
|  |  |  |  |  |
| Secondary outcomes |  |  |  |  |
| Na/K ratio at week 16 in adults | NA | xx.xx (xx.xx to xx.xx) | 0.xxx | 0.xxx |
| SBP in adults* | NA | xx.xx (xx.xx to xx.xx) | 0.xxx | 0.xxx |
| DBP in adults | NA | xx.xx (xx.xx to xx.xx) |  | 0.xxx |
| SBP in adolescents | NA | xx.xx (xx.xx to xx.xx) |  | 0.xxx |
| SBP in adolescents | NA | xx.xx (xx.xx to xx.xx) |  | 0.xxx |
|  |  |  |  |  |

Note: DiD: difference of difference, referring to the average change of values from baseline to week 16 between the two groups (intervention vs control)

*P value after multiple comparison adjustment

Table 11. Protocol violation and deviations

| Event | Potassium-enriched salt substitute  (N = ) | Traditional table salt  (N = ) |
| --- | --- | --- |
| Major (protocol violation) | **nEVT nPT(xx.x%)** | **nEVT nPT(xx.x%)** |
| PV category 1 | nEVT nPT(xx.x%) | nEVT nPT(xx.x%) |
| PV category 2 | nEVT nPT(xx.x%) | nEVT nPT(xx.x%) |
| PV category n | nEVT nPT(xx.x%) | nEVT nPT(xx.x%) |
|  |  |  |
| Minor (protocol deviation) | **nEVT nPT(xx.x%)** | **nEVT nPT(xx.x%)** |
| PV category 1 | nEVT nPT(xx.x%) | nEVT nPT(xx.x%) |
| PV category 2 | nEVT nPT(xx.x%) | nEVT nPT(xx.x%) |
| PV category n | nEVT nPT(xx.x%) | nEVT nPT(xx.x%) |
|  |  |  |

Program note: To be adjusted by data - may delete this if description in text is enough.

## Figures

Figure 1. CONSORT diagram


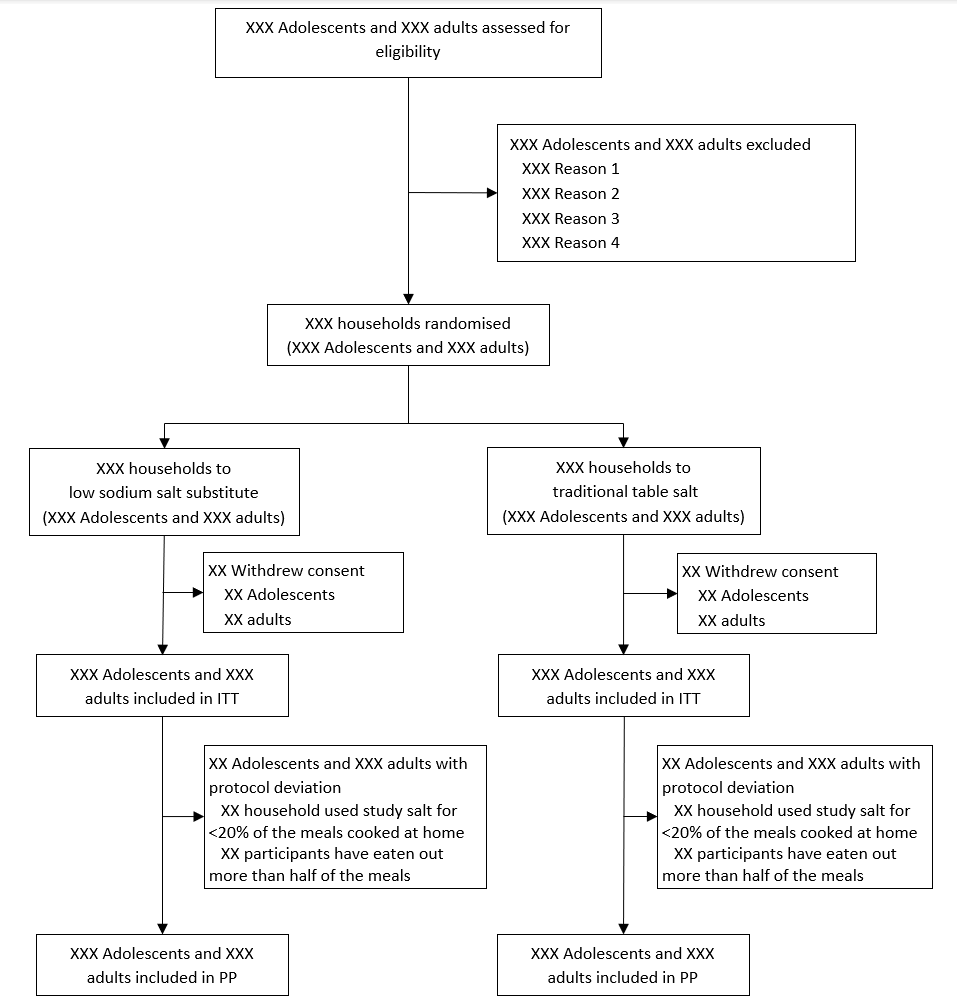


Figure 2. Longitudinal plot of Na/K+ ratio in Adolescents

Note: For Demonstration purpose only

Programming note:

- X-axis will be week 0, 4, 12, 16
- Y axis will be the Na/K+ ratio
- Estimates are raw values (not modelled) with 95% CIs
- Numbers below the plot: numbers of participants with data available
- Add the DiD estimated from the model as a legend

Figure 3. Forest plot for subgroup analysis of change of Na/K ratio at week 16 in adolescents

Programming note: it will be run in ITT dataset using only available data.

The subgroup analyses of change of Na/K ratio at week 16 in adults, change of SBP at week 16 in adults, and change of SBP at week 16 in adolescents may also be shown in forest plots if needed.

Figure 4. Longitudinal plot of Na/K+ ratio in adults

Figure 5. Longitudinal plot of mean SBP and DBP in adults

Figure 6. Longitudinal plot of mean SBP and DBP in adolescents

Programming note: Figure 4-6 are all similar to Figure 2
